# Incomplete Suppression of HIV-1 by SAMHD1 Permits Efficient Macrophage Infection

**DOI:** 10.20411/pai.v3i2.263

**Published:** 2018-12-06

**Authors:** Timothy Plitnik, Mark E. Sharkey, Bijan Mahboubi, Baek Kim, Mario Stevenson

**Affiliations:** 1 Department of Microbiology & Immunology; Miller School of Medicine, University of Miami; Miami, Florida; 2 Department of Medicine; Miller School of Medicine, University of Miami; Miami, Florida; 3 Department of Pediatrics, Emory University; Atlanta, Georgia; 4 Center for Drug Discovery, Children's Healthcare of Atlanta; Atlanta, Georgia; 5 Department of Pharmacy, Kyung-Hee University; Seoul; South Korea

**Keywords:** HIV-1, macrophages, SAMHD1

## Abstract

**Background:**

Sterile alpha motif and histidine/aspartic acid domain-containing protein (SAMHD1) is a dNTP triphosphorylase that reduces cellular dNTP levels in non-dividing cells, such as macrophages. Since dNTPs are required for reverse transcription, HIV-2 and most SIVs encode a Vpx protein that promotes proteasomal degradation of SAMHD1. It is unclear how HIV-1, which does not appear to harbor a SAMHD1 escape mechanism, is able to infect macrophages in the face of SAMHD1 restriction.

**Methods:**

To assess whether HIV-1 had a mechanism to negate SAMHD1 activity, we compared SAMHD1 and dNTP levels in macrophages infected by HIV-1 and SIV. We examined whether macrophages infected by HIV-1 still harbored antiviral levels of SAMHD1 by assessing their susceptibility to superinfection by *vpx*-deleted SIV. Finally, to assess whether HIV-1 reverse transcriptase (RT) has adapted to a low dNTP environment, we evaluated SAMHD1 sensitivity of chimeric HIV-1 and SIV variants in which the RT regions were functionally exchanged.

**Results:**

Here, we demonstrate that HIV-1 efficiently infects macrophages without modulating SAMHD1 activity or cellular dNTP levels, and that macrophages permissive to HIV-1 infection remained refractory to superinfection by *vpx*-deleted SIV. Furthermore, through the use of chimeric HIV/SIV, we demonstrate that the differential sensitivity of HIV-1 and SIV to SAMHD1 restriction is not dictated by RT.

**Conclusions:**

Our study reveals fundamental differences between HIV-1 and SIV in the strategy used to evade restriction by SAMHD1 and suggests a degree of resistance of HIV-1 to the antiviral environment created by SAMHD1. Understanding how these cellular restrictions antagonize viral replication will be important for the design of novel antiviral strategies.

## INTRODUCTION

The replication of primate lentiviruses is antagonized by several cellular factors, collectively referred to as antiviral restrictions. These include APOBEC3G, Tetherin/BST2, and Sterile alpha motif and histidine/aspartic acid domain-containing protein (SAMHD1). Primate lentiviruses have evolved strategies to counteract these restrictions thus allowing infection of, and replication within, host cells. APOBEC3G is countered by the accessory protein Vif, Tetherin/BST2 is neutralized by Vpu, and SAMHD1 is nullified by Vpx which is expressed by HIV-2 and most SIVs. In each case, the viral defense protein targets the cellular restriction for proteasomal destruction [1].

SAMHD1 limits dNTP concentrations in non-dividing cells where high levels of dNTPs would be excessive to need [2]. Non-cycling cells need to maintain low levels of dNTPs to prevent the buildup of cytosolic nucleic acid that could trigger innate immune responses [2, 3]. SAMHD1 is preferentially expressed in macrophages which are an important target cell for primate lentivirus infection as well as dendritic cells and quiescent CD4+ T cells [4]. SAMHD1 is a GTP-activated nucleotide triphosphohydrolase, which converts dNTPs into deoxyribonucleosides and triphosphates [2, 5–7]. SAMHD1 is responsible for the inhibition of a wide range of viruses and retroviruses in myeloid cells [8–10], and defects in SAMHD1 can cause Aicardi-Goutières Syndrome, an autoimmune disorder characterized by persistent immune activation [11, 12]. Most reports agree that SAMHD1 restricts infection through its dNTPase activity, though there are some reports indicating that RNase activity is required for antiviral restriction [13]. The antiviral activity of SAMHD1 is further regulated via its phosphorylation status, being more active in non-cycling cells or after interferon and cytokine exposure [11, 14, 15].

The antiviral action of SAMHD1 is opposed by the lentiviral accessory protein Vpx, which is encoded by HIV-2 and most SIVs but is absent from HIV-1. In the presence of SAMHD1, cellular dNTP concentrations are suboptimal for reverse transcription of viral cDNA [2, 16, 17]. Indeed, for SIV variants lacking a functional *vpx* allele, macrophages are completely refractory to infection [9, 18, 19]. This therefore requires that primate lentiviruses eliminate SAMHD1 for reverse transcription to occur. As such, Vpx is encapsidated in the virion through interaction with Gag p6 [20, 21]. This allows rapid delivery of Vpx upon cytosolic discharge of the viral core. Following fusion of viral and cellular membranes, SAMHD1 is rapidly recruited by Vpx to the DCAF1/Cullin/E3 Ubiquitin Ligase complex and subsequently tagged for proteosomal degradation [2, 7, 22–24]. SAMHD1 can also be eliminated *in trans* through the addition of Vpx-containing virus-like particles (Vpx-VLPs) thereby augmenting macrophage infection by SIV/HIV-2, HIV-1, and other retroviruses [2, 9, 22]. Upon SAMHD1 degradation, intracellular dNTP levels rapidly increase to levels conducive to efficient reverse transcription [7, 22, 25, 26]. Therefore, maintaining low cellular dNTP levels represents a potent mechanism for preventing reverse transcription and viral infection [2, 8, 27, 28].

HIV-1 presents a conundrum with regards to SAMHD1 evasion. HIV-1 encodes a structurally similar Vpx paralog termed Vpr, which likely evolved through duplication of an ancestral *vpx* allele [29–31]. Vpx proteins from different SIV strains exhibit differential binding to SAMHD1, and some SIVs encode Vpr proteins that can bind SAMHD1 [29, 30, 32]. It has been hypothesized that HIV-1 Vpr may have some SAMHD1 degradation properties as it also binds DCAF1 [33, 34]. However, despite similarities between the proteins, HIV-1 Vpr appears unable to substitute for SIV Vpx in enhancing macrophage infection in the presence of SAMHD1 [19].

A number of studies have turned to HIV-1 reverse transcriptase (RT) to possibly explain how HIV-1 can infect macrophages in the face of SAMHD1 restriction. Studies show that the lentiviral RTs are exceptionally good at operating at low dNTP levels, much more so than other retroviruses [35–37]. Several studies have reported that the Km of HIV-1 RT for dNTPs is above the levels found in macrophages [17, 38]. This has prompted investigators to suggest that HIV-1 RT has evolved to operate in a low dNTP environment thus enabling macrophage infection without the need to evade SAMHD1 [39, 40]. Here we investigate the impact of SAMHD1 on permissivity of macrophages to HIV-1 infection. Specifically, we assessed whether HIV-1 promotes SAMHD1 degradation or modulates its antiviral activity, whether HIV-1 modulates dNTP levels, and finally, whether RT influences the ability of HIV-1 to evade SAMHD1 restriction.

## METHODS

### Cell Culture

Elutriated human monocytes from anonymous donors were shipped overnight from the University of Nebraska Medical Center. The cells were further processed with EasySep™ Human Monocyte Isolation Kit (StemCell Technologies) and differentiated for 7 days under standard tissue-culture conditions (37°C, 5% CO_2_) in the presence of recombinant human M-CSF (R&D Systems) at 10 ng/mL in macrophage growth media comprised of 1xDMEM containing 10% heat-inactivated human serum (SeraCare Life Sciences), 1% 200mM L-Glutamine (Gibco), and 0.1% Gentamicin (Sigma). Isolated monocytes were plated in 24-well polypropylene plates at a density of ~0.5-0.75e6 cells/well in 1.5 mL [41]. HEK293T cells were cultured in 1xDMEM containing 10% heat-inactivated FBS (Hyclone), 1% 200mM L-Glutamine (Gibco), and 1% Pen-Strep (Gibco). In addition, sMAGI cells were obtained from the AIDS Reagent Program and cultured as described [42].

### Viral Molecular Clone Generation

The molecular clone, SIVsmm PBj1.9 (SIVwt) and its counterpart lacking a functional *vpx* allele (SIVΔvpx) are as previously described [18]. HIV-1 LAIada is a macrophage tropic HIV-1 variant in which the infectious HIV-1 molecular clone LAI contains a portion of the envelope of macrophage tropic HIV-1 ADA [43]. SIVΔvpx+Vpx, in which Vpx is packaged *in trans*, was generated by co-transfection of the SIVsmm PBjX2 (SIVΔvpx) plasmid with a CMV-driven SIV PBj Vpx expression plasmid. SIVwt-GFP and SIVΔvpx-GFP are clones of PBj that contain a GFP gene inserted in place of Env [19]. HIV-1-GFP contains a GFP gene inserted in Nef [19]. The chimeric virus RT-SHIV (in which HIV-1 RT from LAI is inserted in an SIV PBj backbone in place of SIV RT) was generated using Exponential Megapriming PCR [44] and Q5 High-Fidelity DNA Polymerase (New England Biolabs). Primers used were as follows: SHIV-F1-CCCATTAGTCCTATTGAAACTGTACCAGTA; SHIV-R1-GCGCTGGTTCTATTTTTTCTAAGAATAGTACTTTCCTGATTCCAGACGT; SHIV-R2-GAGATTTAGAGACATACCCATAGCTGTTAG. The following primers were used to generate RT-HSIV, (in which the RT from SIV PBj is inserted into an HIV-1 LAI backbone): HSIV-F1-CCCATAGCTAAGGTAGAGCCTATAAAAGTA; HSIV-R1-GGGCCTTATCTATTCCATCTAAAAATAGGACTTGTCTAATTCCTTGACTA; HSIV-R2-AAAATTTAAAGTGCAACCAATCTGAGTCAA. Full-length SIVmac316e WT and Δvpx were generated by combining plasmids containing the 5' end of SIVmac239 (wild type and Δvpx) with the 3' end of SIVmac316e. RT-SHIVmac316e wild type and Δvpx were generated by recombining the RT containing region of a RT-SHIVmac239 plasmid with the respective full-length SIVmac316e plasmids. All SIV/RT-SHIVmac plasmids were kindly provided by the laboratory of Ronald C. Desrosiers, PhD at the University of Miami [45–47].

### Generation of Viral Stocks

Infectious virus stocks were generated by Lipofectamine^®^ 2000 (Thermo Fisher) transfection of HEK293T cells. Vesicular Stomatitis Virus glycoprotein (VSVg)-transcomplementation was used with HIV-1, SIV, RT-SHIV, and RT-HSIV clones to increase the infectivity of the viral stocks [48, 49]. A plasmid expressing SIVsmm PBj Vpx was also co-transfected to generate SIVs with Vpx added *in trans*. Transfected cell supernatants were harvested 48 and 72 hours post-transfection, concentrated over a 20% sucrose gradient by ultra-centrifugation, and stored at -80°C until use. Viral stocks were quantified by commercially available p24 or p27 ELISAs for HIV-1 and SIV preparations respectively (Advanced Bioscience Laboratories).

### Macrophage Infection

Macrophages in 24-well plates were infected (50 ng of p24 or p27 per well) for 4-6 hours at 37°C, after which cells were washed with PBS, and virus-containing media was replaced with fresh media. In some cases, spinoculation (centrifugation at 1200*g* for 2 hours at 25°C) was also used to increase viral infectivity. At specified time points post-infection, total DNA was extracted using ZR-96 Quick-gDNA™ kits (Zymo Research). To gauge the contribution of carryover plasmid DNA to the cDNA measurements, antiretroviral drugs were added at supra-inhibitory concentrations (400µM Tenofovir or 2µM Nevirapine) at least 2 hours before infection and maintained throughout the infection.

### SAMHD1 Knock-Down

Macrophages were transfected with 60 pmol SAMHD1-specific siRNA using GenMute™ siRNA Transfection Reagent for Primary Macrophages (SignaGen Laboratories) according to the manufacturer's instructions. Cells were again transfected on day 4 and day 8 after the initial transfection (total of 3 transfections). Cells were ready for infection or lysis 14 days after the initial transfection. The following siRNAs were used: SAMHD1 silencer select siRNA (Thermo Fisher Scientific); Silencer^®^ Select Negative Control No. 1 siRNA (Life Technologies). Lyophilized siRNAs were re-hydrated in 1x siRNA Buffer (Dharmacon) at a concentration of 20nM and stored at -20°C.

### Western Blots

Cells were lysed using RIPA Lysis and Extraction Buffer (Life Technologies) with Halt™ Protease and Phosphatase Inhibitor Cocktail (Life Technologies). Total protein was quantified by EZQ™ Protein Quantitation Kit (Thermo Fisher Scientific). Lysates were denatured using NuPAGE^®^ LDS sample buffer and reducing reagent (Life Technologies). Samples were run on NuPAGE^®^ Bis-Tris Gels (Life Technologies) and transferred to a nitrocellulose membrane using iBlot^®^ Transfer Stack (Life Technologies). Antibodies used were as follows: Anti-SAMHD1 antibody, clone 3F5 (Abcam); GAPDH antibody, clone 2D9 (Origene). MagicMark™ XP Western Protein Standard (Life Technologies) was used as a molecular weight standard. Densitometry analysis was performed using ImageJ 1.50i software.

### Quantification of Reverse Transcripts

Levels of 2LTR circles, a marker of complete reverse transcription, were determined using TaqMan^®^ Fast Universal PCR Master Mix (Life Technologies) on an Applied Biosystems^®^ 7500 Fast Real-Time PCR System. In each 20 µL reaction (performed in duplicate) 5 µL of total DNA was used. The 2LTR circles were normalized to CCR5 copy number (cell equivalents). Samples were quantified using standard curves of known quantities of each amplicon. Primer/Probes sets were as follows: CCR5: CCR5F- GCTGTCTTTGCGTCTCTCCCAGGA; CCR5R- CTCACAGCCCTGTGCCTCTTCTTC; CCR5probe- /56FAM/AGCAGCGGC/ZEN/AGGACCAGCCCCAAG/3IABkFQ/; SIV: PBjn/f- AGAAGCCCCTGGTCTGTTAGGAC; PBjc/r- TCGTCTTCCTGAGCTTCATCTGA; PBjprobe- /56-FAM/TGGCAAAAT/ZEN/TACACAGCAGGGCCAG/3IABkFQ/; HIV-1: HIV1F- TAGACCAGATCTGAGCCTGGGA; HIV1R- GTAGTTCTGCCAATCAGGGAAG; HIV1probe- /56FAM/AGCCTCAAT/ZEN/AAAGCTTGCCTTGAGTGC/3IABkFQ/.

### Flow Cytometry

Macrophages were stained with LIVE/DEAD^®^ Fixable Near-IR Dead Cell Stain Kit (Thermo Fisher) and fixed in 4% paraformaldehyde. They were then detached with Accutase™ Cell Detachment Solution (StemCell Technologies™), permeabilized with an NP-40 solution, blocked with Human TruStain FcX™ solution (Biolegend), and stained with anti-HIV-1 Core Antigen, Clone: KC57-RD1 (Beckman Coulter™) and Alexa-488 Anti-SAMHD1 (I-1918) antibody, kindly provided by the Olivier Schwartz at the Pasteur Institute [26]. Data was analyzed on FlowJo v10.1 software.

### dNTP Assay

For dNTP extraction, cells were lysed in 65% ice-cold methanol. Lysates were then vortexed for 2 minutes, heated to 95°C for 3 minutes, cooled on ice for 2 minutes, and subsequently centrifuged to clear debris. Cleared lysates were then dried using a speed-vac at 50°C. The dried lysates were frozen at -80°C until analysis. The dNTP concentrations were determined by the Baek Kim Laboratory at Emory University as previously described [17].

### RT-Assay

After infection, aliquots of supernatant were harvested at several time-points and frozen at -20°C until ready to be assayed using a previously established protocol [50]. Briefly, frozen supernatant was thawed, and 5 µL was incubated for 15 minutes at 37°C in a 96-well round-bottom plate with 10 µL of Solution A (100mM Tris-HCl, 300mM KCl, 10mM DTT, 0.1% NP40) to lyse the virus. Next, 25 µL of Solution B (50mM Tris-HCl, 150mM KCl, 5mM DTT, 0.5% NP40, 15mM MgCl_2_, 0.5 U/mL PolyA-Oligo dT, and 30 µCi ^3^H-dTTP) was added to each well, and the plate was incubated at 37°C overnight. The next day, 5 µL of the reaction was spotted onto Whatman DEAE filter paper, washed 3 times with 5% Na_2_HPO_4_, washed with DI water, washed with 70% Ethanol, dried, and read in a scintillation counter for 60 seconds.

### Statistics

Data was analyzed on Prism 7 software. Data comparisons between 2 experimental groups were analyzed by an unpaired *t* test with Welch's correction or by area under the curve analysis. *P* values are denoted as *P<0.05, **P<0.01, ***P<0.001, and ****P<0.0001. All graphs, unless otherwise stated, represent pooled data from at least 3 or more separate, replicate experiments +/− SD.

## RESULTS

### SAMHD1 is Modulated by SIV, But Not HIV-1 Infection.

**Figure 1.**
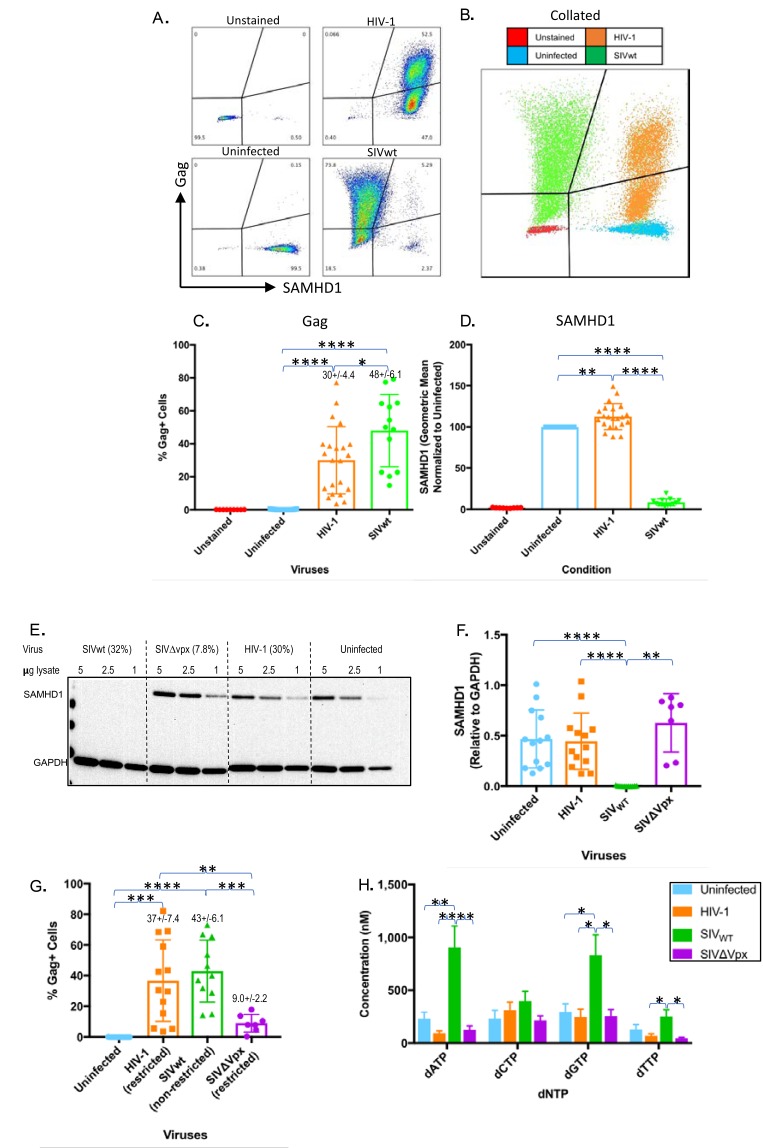
**SIV but not HIV-1 eliminates SAMHD1 and augments cellular dNTP levels in macrophages.** (A-B) FACS analysis of viral Gag and SAMHD1 levels in uninfected macrophages and in macrophages infected with HIV-1 or SIV. Individual FACS profiles together with combined profiles are indicated in panel A and B respectively. (C-D) Quantitative (geometric means) and statistical comparisons of viral Gag (C) and SAMHD1 (D). (E) Western blot analysis (cropped image) of SAMHD1 levels in uninfected, HIV-1, SIVwt, and SIVΔvpx-infected macrophages together with quantitative and statistical analysis of SAMHD1 levels (F) are indicated. Lysates for Western blotting and intracellular dNTP analysis (H) were generated 1 day post-infection. Cells from the same infection were sampled 4 days post-infection for intracellular Gag staining. (G) The percentage of viral Gag-positive cells from the same experiments as samples collected for Western blotting and dNTP measurements and above the Western blot for that particular experiment. Results represent pooled data from 3 or more replicate experiments +/− SD. Welch's t-test: *P < 0.05 **P < 0.01 ***P < 0.001 ****P < 0.0001.

The lack of a Vpx-like function in HIV-1 would prompt speculation that macrophages are refractory to HIV-1 infection. Therefore, we first assessed whether HIV-1 can infect macrophages as robustly as SIV. Monocyte-derived macrophages were infected with wild-type HIV-1 or SIV (HIV-1 or SIVwt) that were transcomplemented with VSVg. Spinoculation was further employed to promote efficient and synchronous infection of macrophages [28, 48]. Three to 4 days after infection, cells were analyzed for co-expression of HIV-1 or SIV Gag and SAMHD1 by flow cytometry (Figure 1A, B). Individual profiles and collated profiles are shown in A and B respectively. As expected, SIV Gag-positive macrophages expressed very low levels of SAMHD1, while in contrast, HIV-1 Gag-positive cells expressed levels of SAMHD1 similar to those in uninfected cells (Figure 1A, B). Statistical comparison of Gag and SAMHD1 levels in SIV- and HIV-1-infected cells (Figure 1C, D) confirmed that not only do both HIV-1 and SIV infect macrophages at comparable levels (30% +/− 4.4% for HIV-1 and 48% +/− 6.1% for SIV), but only SIV infection of macrophages led to elimination of SAMHD1. HIV-1 infection, however, did not alter SAMHD1 levels in macrophages as drastically, and the levels were more comparable to those seen in uninfected cells (Figure 1D). The slightly higher SAMHD1 levels observed in HIV-1-infected macrophages are likely due to spectral overlap between the fluorochromes (PE and Alexa488).

To confirm the flow cytometry results, we next examined SAMHD1 levels in infected cells by Western blotting. Western blots of infected macrophage lysates revealed a similar pattern of SAMHD1 degradation in SIVwt-infected but not HIV-1-infected or *vpx*-deleted SIV (SIVΔvpx)-infected macrophages (Figure 1E, F). In SIVwt-infected macrophages, SAMHD1 was not detectable (Figure 1E, F). In contrast, SAMHD1 levels in HIV-1-infected cells were comparable to those in uninfected cells despite similar levels of Gag+ cells from wells sampled for flow cytometry (37% +/− 7.4% for HIV-1 and 43% +/− 6.1% for SIVwt) (Figure 1F, G). Not surprisingly, SIVΔvpx did not affect SAMHD1 levels and was restricted from infection (Figure 1E, F, G). Densitometry demonstrated no significant differences in SAMHD1 levels between HIV-1-infected, SIVΔvpx-infected, and uninfected macrophages (Figure 1F).

Hypothetically, it was possible that HIV-1 used a mechanism, independent of SAMHD1 degradation, to augment dNTP levels and create conditions conducive to reverse transcription. Therefore, we measured intracellular dNTP levels in HIV-1- and SIV-infected macrophages. As expected, infection of macrophages with SIV led to an increase in dNTP levels, in particular dATP and dGTP, compared with levels in uninfected cells (Figure 1H). In contrast, dNTP levels in HIV-1-infected and SIVΔvpx-infected macrophages were similar to those in uninfected macrophages (Figure 1H). Collectively, these results indicate that HIV-1 undergoes macrophage infection without modulating cellular SAMHD1 or dNTP levels. The results also confirm previous findings that SIV is dependent on SAMHD1 depletion and dNTP modulation [7, 8, 19, 23, 51]. This suggests that HIV-1 may exhibit some degree of intrinsic resistance to the antiviral environment created by SAMHD1.

### SAMHD1 Knock-Down Impacts Macrophage Permissivity to Lentiviral Infection

**Figure 2.**
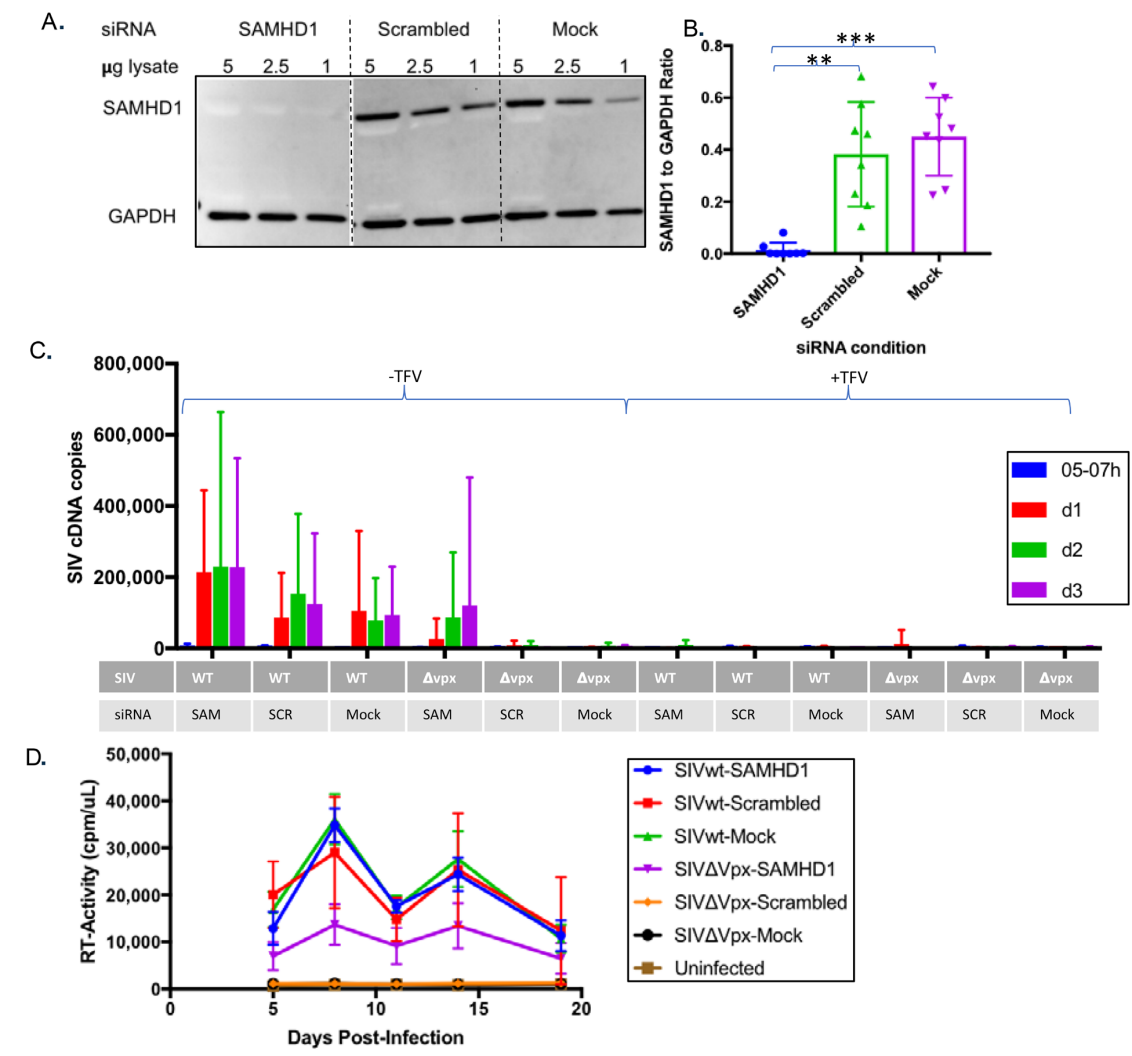
**SAMHD1 knock-down renders macrophages permissive to infection by** ***vpx*****-deleted SIV.** (A) Complete elimination of SAMHD1 expression in macrophages through successive (3X) rounds of siRNA transfection. Levels of SAMHD1 in macrophages 14 days after transfection with SAMHD1-specific or scrambled siRNAs or mock-transfected macrophages are shown by Western blot (cropped image). (B) SAMHD1 levels were quantitated by densitometry for statistical comparisons of SAMHD1 as normalized to GAPDH levels. (C-D) SAMHD1 knock-down permits macrophage infection by SIVΔvpx. Fourteen days after siRNA transfection, macrophages were infected with wild-type (wt) or *vpx*-deleted (SIVΔvpx) variants, and infection of siRNA transfected macrophages was gauged from the levels of full-length viral cDNA by qPCR analysis (C) and by viral reverse transcriptase activity in culture supernatants (D) at the indicated intervals post-infection. Bar graphs represent pooled data from 3 or more replicate experiments +/− SD. Welch's t-test: *P < 0.05 **P < 0.01 ***P < 0.001 ****P < 0.0001.

To further confirm the antiviral effects of SAMHD1, we examined the effect of SAMHD1 depletion on macrophage infectivity. SAMHD1 has a long half-life, so we developed a triple siRNA transfection strategy to fully deplete SAMHD1 levels in primary macrophages [22, 52]. Unlike other studies that rely on monocytic cell-lines or partial knockdown, our strategy was effective in depleting SAMHD1 in primary human monocyte-derived macrophages to levels undetectable by Western blotting (Figure 2A) [2, 22, 23, 53–57]. Densitometry analysis confirmed that the SAMHD1-targeting siRNA led to depletion of SAMHD1 to levels below both those of scrambled siRNA (*P*<0.01) and mock transfection (*P*<0.001) (Figure 2B). SAMHD1-depleted macrophages were then infected with SIVwt or SIVΔvpx, and infection was monitored by the level of viral cDNA (2LTR circles) by qPCR. Although 2LTRs are unintegrated episomal products of reverse transcription, they nonetheless serve as convenient indicators of viral infection under single-round conditions [58]. To demonstrate that cDNA products detected in infected macrophages were synthesized *de novo*, macrophages were infected in the presence and absence of supra-inhibitory concentrations of the RT inhibitor tenofovir. While the scrambled siRNA and mock transfection conditions had little to no detectable viral cDNA for SIVΔvpx, depletion of SAMHD1 mediated by SAMHD1-siRNA increased viral cDNA levels in SIVΔvpx-infected macrophages comparable to those observed in wild-type SIV-infected macrophages (Figure 2C). Viral cDNA products were barely detectable in macrophages infected in the presence of tenofovir (Figure 2C). This result was further confirmed by an increase in productive infection as evidenced by levels of RT activity in culture supernatants (Figure 2D). SAMHD1 knock-down treatment significantly increased SIVΔvpx viral production above its scrambled and mock conditions. These results confirm that SAMHD1 depletion is necessary and sufficient to compensate for the macrophage infectivity defect created by *vpx* deletion, even though Vpx has functions besides SAMHD1 degradation [18, 59, 60].

### HIV-1 Does Not Alter the Antiviral Environment Created by SAMHD1

**Figure 3.**
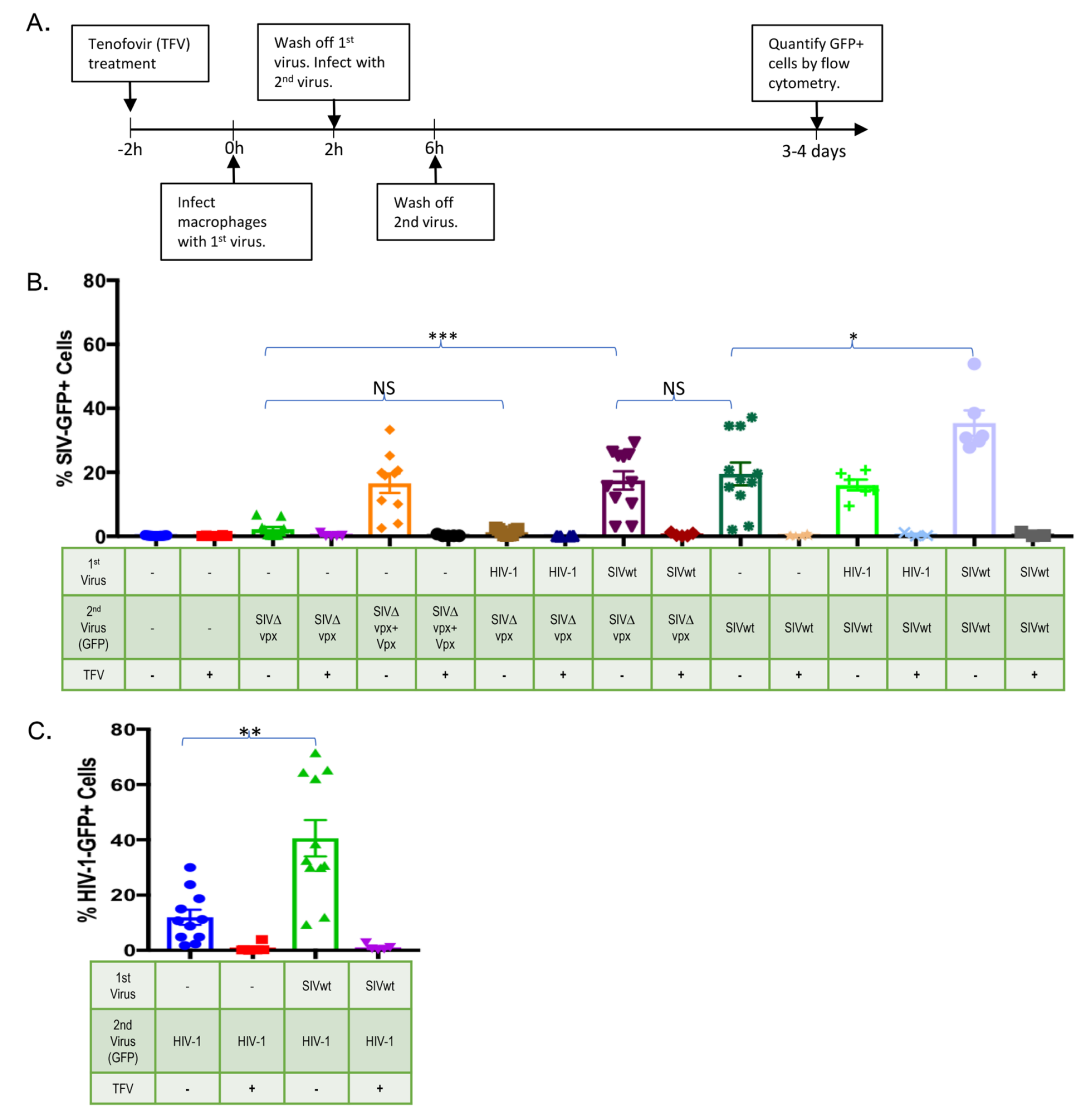
**HIV-1 preinfection does not render macrophages permissive to superinfection by** ***vpx*****-deleted SIV.** (A) Experimental design. Macrophages were first infected with HIV-1 or SIV and after 2 hours, superinfected with wild-type (SIVwt) or *vpx*-deleted (SIVΔvpx) SIV variants expressing GFP. (B) Macrophage infection by wild-type and *vpx*-deleted SIV variants was gauged from the number of GFP positive cells at 3-4 days post-infection. To gauge possible contribution of macrophage autofluorescence to the GFP signal and to ascertain *de novo* infection, tenofovir, which inhibits HIV-1 and SIV reverse transcriptases, was added to duplicate cultures 2 hours prior to infection. (C) SIVwt pre-infection also increased macrophage permissivity to HIV-1-GFP infection. Bar graphs represent pooled data from 3 or more replicate experiments +/− SD. Welch's t-test: *P < 0.05 **P < 0.01 ***P < 0.001 ****P < 0.0001.

Although HIV-1 appeared capable of infecting macrophages without altering SAMHD1 or dNTP levels, there remained the possibility that HIV-1 harbors an unknown function to nullify the anti-viral environment. If this were the case, one would predict that macrophages infected with HIV-1 would exhibit increased permissivity to superinfection by *vpx*-deleted SIV. Macrophages were first infected for 2 hours with either HIV-1 or SIVwt and were then super-infected with a GFP-expressing HIV-1, SIVwt, or SIVΔvpx. Three to 4 days after infection, the frequency of GFP-positive macrophages was assessed by flow cytometry (Figure 3A). As expected, macrophages were completely refractory to SIVΔvpx infection, and prior infection with SIVwt normalized SIVΔvpx infection of macrophages to SIVwt levels (*P*<0.001) (Figure 3B). The susceptibility of macrophages to SIVΔvpx infection was also markedly augmented (*P*<0.001) by loading SIVΔvpx with Vpx *in trans* (SIVΔvpx +Vpx) as evidenced by GFP-positive cells (Figure 3B). In contrast, pre-infection of macrophages with HIV-1 did not augment SIVΔvpx infection of macrophages based on GFP levels (Figure 3B). HIV-1 pre-infection was not able to alter the intracellular environment in a way that would allow reverse transcription of SIVΔvpx; only the addition of Vpx *in trans* could bring SIVΔvpx cDNA to levels seen with wild-type SIV infection (Figure 3B). While macrophages were already infectable by HIV-1-GFP, that infectivity was increased (approximately 3 fold) when macrophages were pre-infected with SIVwt (*P*<0.01 ) (Figure 3C). However, an increase was also observed when macrophages were first infected with SIVwt and super-infected with SIVwt-GFP (*P* < 0.05) (Figure 3B). Collectively, these results indicate that HIV-1-infected macrophages remain restricted to infection by *vpx*-deleted SIV, and that HIV-1 infects macrophages without altering the SAMHD1 antiviral environment that otherwise completely restricts *vpx*-deleted SIV.

### Construction and Validation of RT-SHIV.

**Figure 4.**
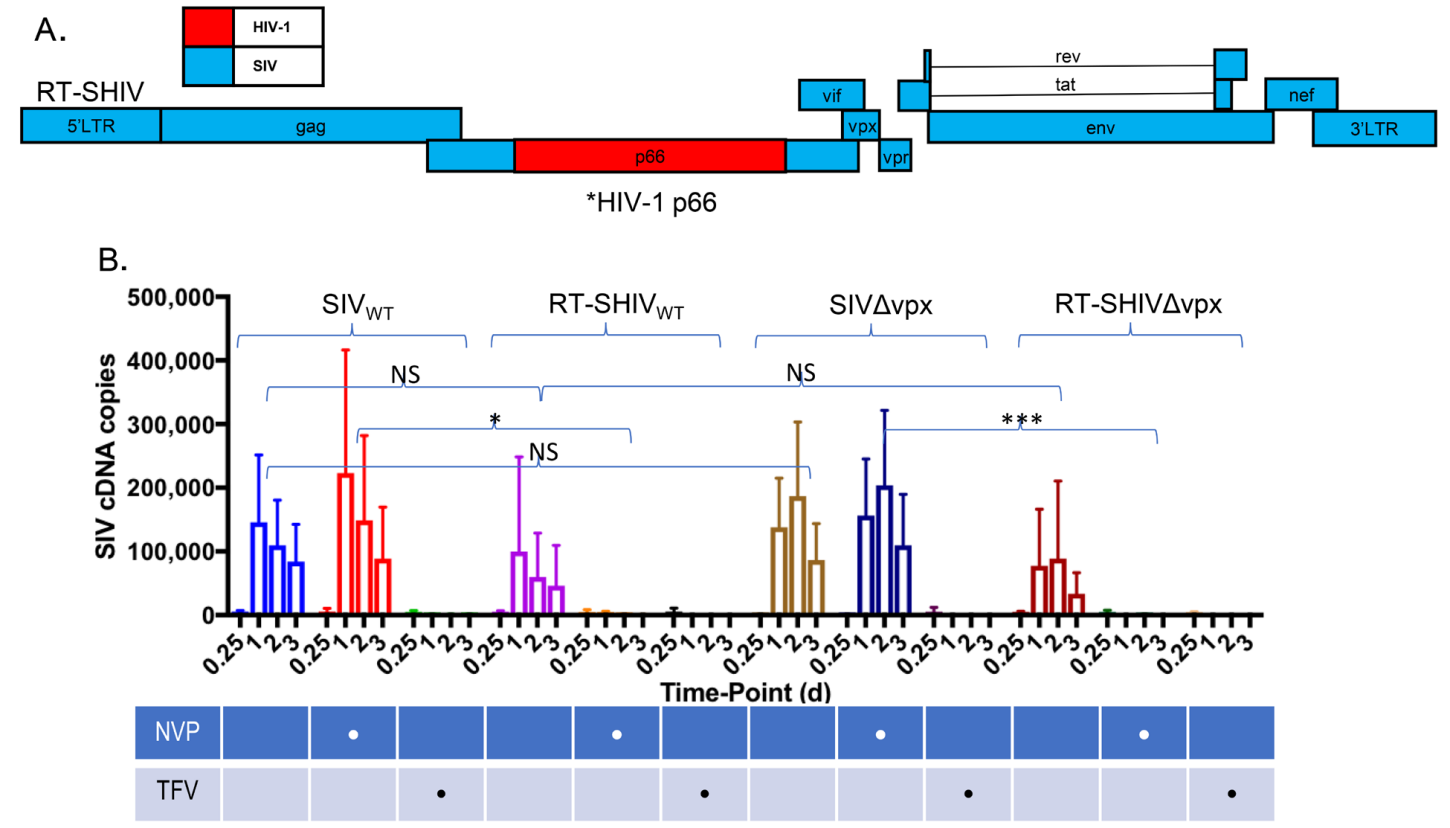
**Construction and validation of RT-SHIV in which HIV-1 RT is inserted into an SIV backbone.** (A) schematic of RT-SHIV in which the HIV-1 reverse transcriptase was inserted into an SIV backbone. (B) differential sensitivity of RT-SHIV to the RT inhibitors nevirapine (NVP) and tenofovir (TFV) was assessed in sMAGI cells (susceptible to infection by HIV-1 and SIV). Tenofovir is active against HIV-1 and SIV RT while nevirapine is only active against HIV-1. sMAGI cells were pretreated with the RT inhibitors for 2 hours and then infected with wild type or *vpx*-deleted SIV variants or with RT-SHIV variants harboring a functional (RT-SHIVwt) or inactive *vpx* (RT-SHIVΔvpx) open reading frame. Infection of sMAGI cells was gauged from levels of viral cDNA by qPCR at the indicated intervals post-infection. Results represent pooled data from 3 or more replicate experiments +/− SD. *P < 0.05 **P < 0.01 ***P < 0.001 ****P < 0.0001.

The data outlined above indicate that HIV-1 infection of macrophages occurs despite SAMHD1 restriction and suggest that this ability might be intrinsic to HIV-1 itself. It has been proposed that the differential sensitivity of HIV-1 and SIV to SAMHD1 is dictated by the Km of their reverse transcriptases, such that the Km of HIV-1 RT allows more efficient dNTP incorporation. [7, 17, 37, 40]. This would predict that exchange of HIV-1 and SIV reverse transcriptases should exchange the sensitivity to SAMHD1 restriction. Therefore, we constructed RT-SHIV, a chimeric virus in which the SIV RT gene was replaced by that of HIV-1. Briefly, EMP-PCR was used to clone the p66 region of HIV-1 into a wild-type or a *vpx*-deleted SIV backbone (SIVsmm PBj) to create RT-SHIVwt and RT-SHIVΔvpx, respectively (Figure 4A). We first validated the functionality of the RT-SHIVs by assessing their sensitivity to nucleoside (NRTIs) and non-nucleoside reverse transcriptase inhibitors (NNRTIs) in the indicator cell line sMAGI, which is infectable by both HIV-1 and SIV [42]. HIV-1 RT is sensitive to both nevirapine and tenofovir, whereas SIV RT is sensitive to tenofovir but insensitive to nevirapine [61]. The infectivity of the RT-SHIVs in sMAGI cells, as assessed by qPCR for viral cDNA, was comparable to that observed for the parental SIVs (Figure 4B). While SIV infection was insensitive to nevirapine, no cDNA products were observed in RT-SHIVwt or RT-SHIVΔvpx-infected cells in the presence of nevirapine (Figure 4B). Furthermore, cDNA synthesis by both SIV and RT-SHIV was inhibited by tenofovir demonstrating that cDNA products were generated *de novo*. The ability of nevirapine to inhibit RT-SHIV but not SIV infection demonstrated that the RT-SHIVs contained a functional HIV-1 RT in an SIV backbone.

### Reverse Transcriptase Does Not Dictate Sensitivity to SAMHD1

**Figure 5.**
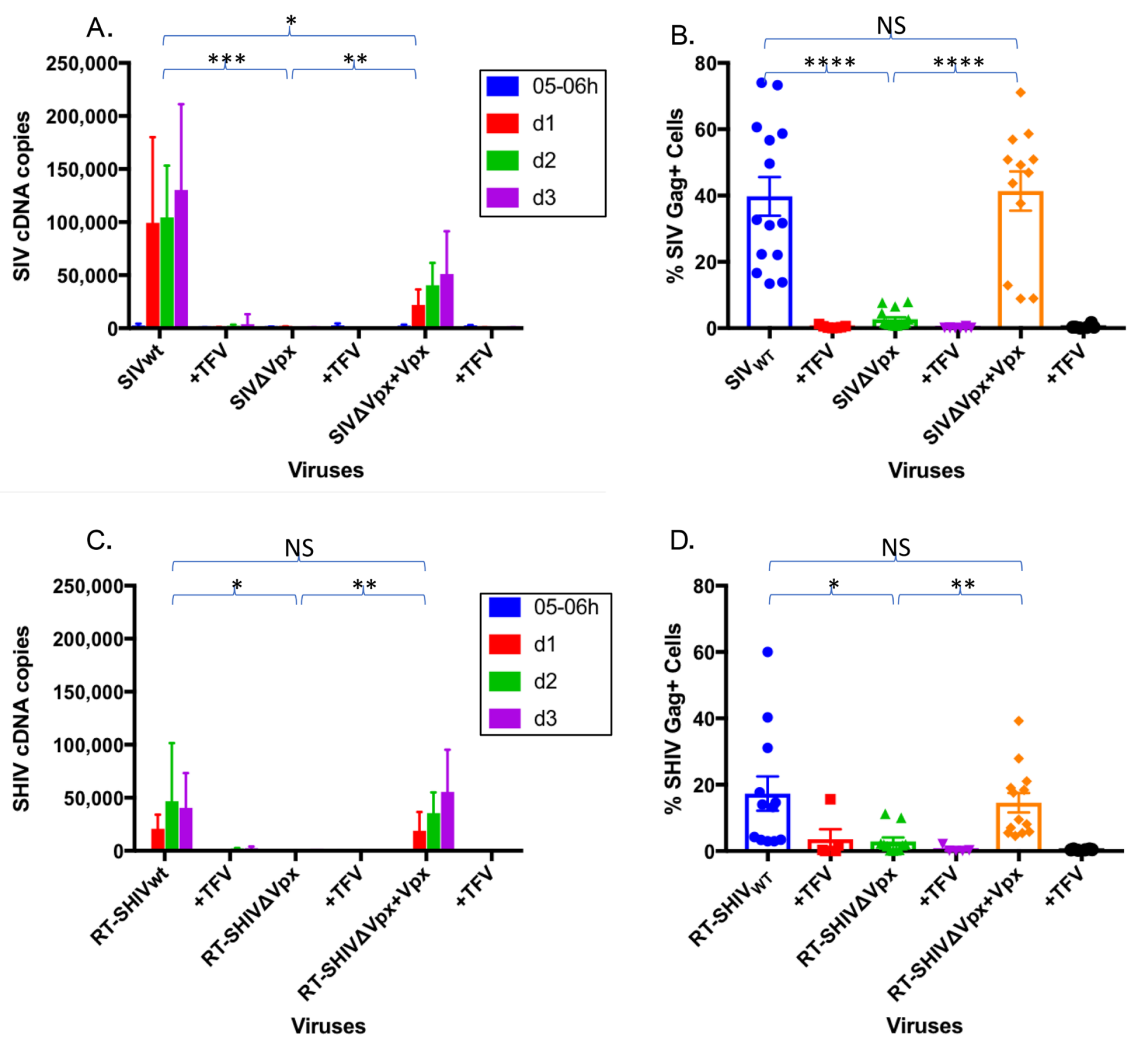
**HIV-1 RT, within the context of an SIV backbone, requires Vpx for reverse transcription in macrophages.** (A-B) Permissivity of macrophages to SIV infection is absolutely dependent on Vpx. Macrophages were infected with wild type or *vpx*-deleted SIV variants and infection was gauged by levels of viral cDNA by qPCR (A) or Gag-positive cells by FACS (B). Introduction of Vpx *in trans* (SIVΔvpx + Vpx) permitted macrophage infection by *vpx*-deleted SIV. (C-D) Permissivity of macrophages to RT-SHIV infection is absolutely dependent on Vpx. Macrophages were infected with RT-SHIV variants containing intact (RT-SHIVwt) or defective (RT-SHIVΔvpx) *vpx* open reading frames and infection gauged by qPCR for viral cDNA (C) and FACS for Gag-positive cells (D). As with parental SIVΔvpx, introduction of Vpx *in trans* rescued *vpx*-defective RT-SHIV (RT-SHIVΔvpx+Vpx). Bar graphs represent pooled data from 3 or more replicate experiments +/− SD. Statistical comparisons for panels A and C are based on Area Under the Curve analysis, and on Welch's t-test for panels B and D) *P < 0.05 **P < 0.01 ***P < 0.001 ****P < 0.0001.

We next evaluated whether the chimeric SIV harboring HIV-1 RT (RT-SHIVwt) and its *vpx*-deleted counterpart (RT- SHIVΔvpx), exhibited reduced sensitivity to SAMHD1 restriction. If indeed HIV-1 RT is capable of operating in a low dNTP environment, we would predict that Vpx would be dispensible for macrophage infection by RT-SHIV. Macrophages were infected with SIV and RT-SHIV variants, and infection was monitored by cDNA production (Figure 5A, B) and by the presence of SIV Gag-positive cells by flow cytometry (Figure 5C, D). Infectivity was compared to wild-type and *vpx*-deleted SIV (SIVwt and SIVΔvpx, respectively). The RT-SHIV harboring an intact *vpx* gene (RT-SHIVwt) was infectious in macrophages as evidenced by *de novo* cDNA generation (Figure 5C) and Gag-positive cells (Figure 5D), albeit at slightly lower levels than SIVwt. In contrast, both SIVΔvpx and RT- SHIVΔvpx were not infectious in macrophages as visualized by cDNA levels (Figure 5A, C) and by numbers of Gag-positive cells (Figure 5B, D). The infectivity of both SIVΔvpx and RT- SHIVΔvpx in macrophages was restored when Vpx was packaged in these viruses *in trans*, as evidenced by cDNA synthesis (Figure 5A, C) and Gag-positive cells (Figure 5B, D). The results were obtained using an HIV RT inserted in a SIVsmm PBj backbone, and we further confirmed these results using chimeric viruses in which HIV-1 RT was inserted into an SIVmac 316e backbone. The wild-type and *vpx*-deleted chimeras were similarly functional as shown by differential sensitivity to nevirapine (Supplemental Figure A). Furthermore, in macrophages, the chimeras were only capable of undergoing reverse transcription in the presence of Vpx (Supplemental Figure B). Collectively, these results demonstrate that chimeric SIVs harboring a functional HIV-1 RT are nevertheless restricted by SAMHD1 and dependent on Vpx for infection of macrophages. This also indicates that reverse transcriptase does not solely account for the ability of HIV-1 to evade SAMHD1 restriction.

### SIV Reverse Transcriptase Does Not Confer Increased Sensitivity of HIV-1 to SAMHD1

**Figure 6.**
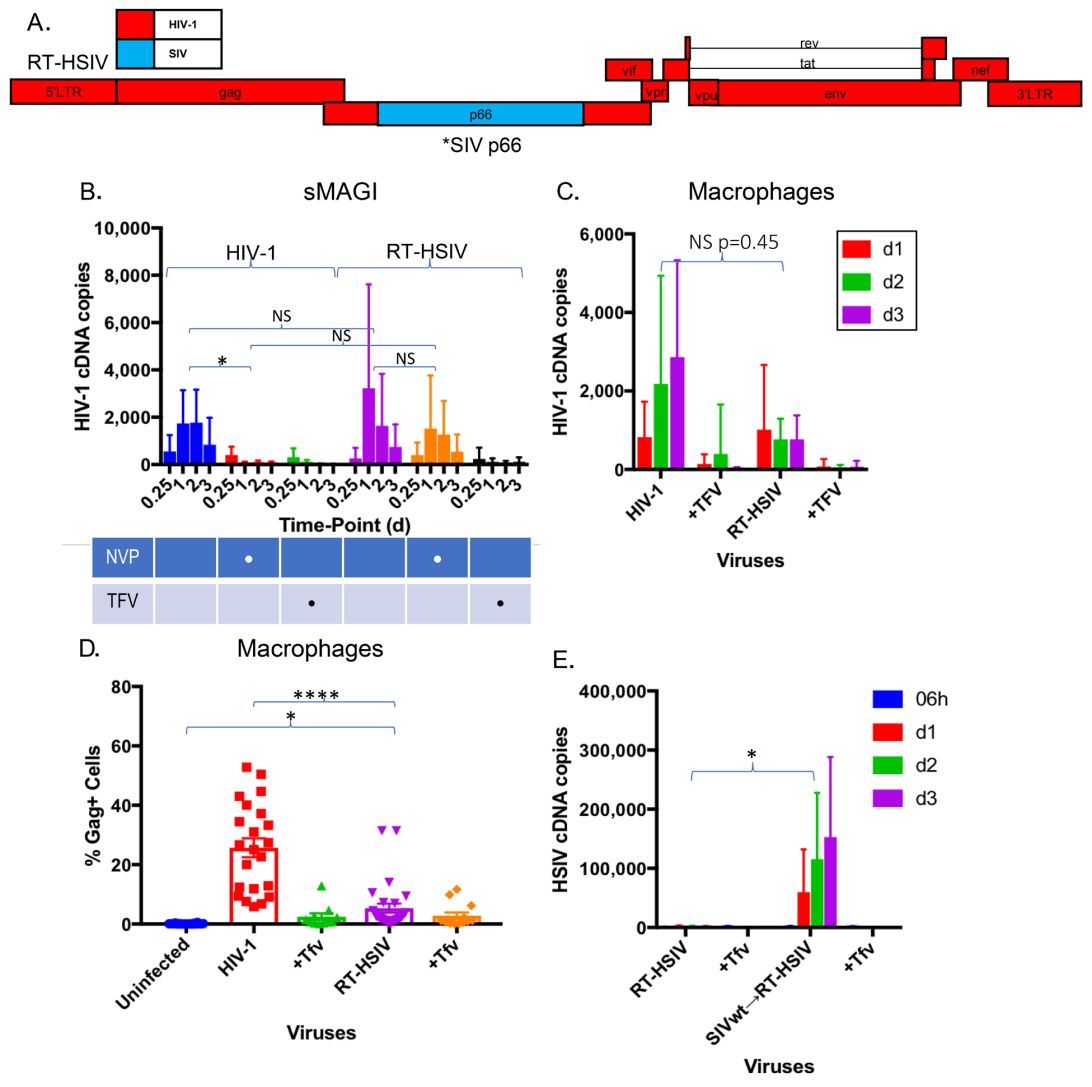
**SIV RT, within the context of an HIV-1 backbone, does not require Vpx for reverse transcription in macrophages.** (A) Schematic of RT-HSIV depicting HIV-1 harboring an SIV RT. (B) RTHSIV infection of sMAGI cells is sensitive to inhibition by tenofovir but not nevirapine. sMAGI cells were infected with wild type HIV-1 (HIV-1) or HIV-1 harboring SIV RT (RT-HSIV) and viral cDNA synthesis assessed by qPCR at the indicated intervals post-infection. (C) Macrophages are equally permissive to infection by wild type HIV-1 and HIV-1 containing SIV RT (RT-HSIV) despite the absence of Vpx. Macrophage infection was assessed by qPCR for viral cDNA at the indicated intervals post-infection. (D) Levels of Gag-positive cells were assessed by flow cytometry after HIV-1 or RT-HSIV infection. (E) Macrophages were infected for 2 hours with SIVwt, then superinfected with RT-HSIV. RT-HSIV cDNA levels were measured by qPCR. Bar graphs represent pooled data from 3 or more replicate experiments +/− SD. Statistical comparisons for panels B, C, and E are based on Area Under the Curve analysis. Panel D statistical comparisons were based on Welch's t-test. *P < 0.05 **P < 0.01 ***P < 0.001 ****P < 0.0001.

Although HIV-1 RT did not appear to confer resistance to SAMHD1 restriction, we wondered if the SIV RT might be inherently more susceptible to low dNTP levels, thus conferring increased sensitivity to SAMHD1. To assess this, we constructed the reciprocal chimeric virus RT-HSIV (Figure 6A); again, EMP-PCR was used to clone the p66 region of SIVsmm PBj into a HIV-1 backbone (HIV-1 LAIada). RT-HSIV was infectious in sMAGI cells as evidenced by *de novo* cDNA generation (Figure 6B). Furthermore, while HIV-1 was sensitive to nevirapine, the RT-HSIV chimera was insensitive to nevirapine (Figure 6B). As expected, both wild-type HIV-1 and the RT-HSIV were sensitive to tenofovir (Figure 6B). These data suggest RT-HSIV contains a functional SIV RT in a HIV-1 backbone. We next assessed whether the chimeric HIV-1 containing SIV RT would exhibit increased sensitivity to SAMHD1 restriction. If differences in efficiency of dNTP usage underscored the differential sensitivity of SIV and HIV-1 to SAMHD1 restriction, we would predict that SIV RT within an HIV-1 backbone would increase the sensitivity of this chimeric virus to SAMHD1. Following macrophage infection, Gag-positive cells were present for HIV-1 (25%+/− 3.2) and at lower levels for RT-HSIV (5.4%+/−1.5) (Figure 6D). However, the level of cDNA products generated with wild-type HIV-1 and the chimeric RT-HSIV were comparable (Figure 6C) and tenofovir substantially reduced cDNA synthesis demonstrating that the cDNA products generated in the absence of tenofovir were bona fide products of reverse transcription (Figure 6C). This was surprising because in the absence of Vpx, SIV RT is incapable of reverse transcribing in macrophages (see, for example, Figure 3C, and 5A). Since the RT-HSIV had the capacity to reverse transcribe in macrophages in which SAMHD1 was active, this suggests that the HIV-1 genome harbors a determinant, apart from RT, enabling SIV RT to operate in the low dNTP environment created by SAMHD1. It is unfortunate that despite reverse transcription, RTHSIV does not produce as much Gag in infected macrophages as HIV-1 (Figure 6D); like many chimeric viruses, this construct may have unforeseen issues with viral fitness unrelated to SAMHD1 restriction [47, 62]. In order to observe the impact of SAMHD1 on RT-HSIV, we pre-infected macrophages with SIVwt for 2 hours and then superinfected them with RT-HSIV. As expected, the Vpx from SIVwt greatly boosts the RT-HSIV cDNA levels (Figure 6E); based on qPCR alone, it would appear that RT-HSIV is quite dependent upon SAMHD1 elimination. However, this phenotype is also observed with HIV-1 cDNA levels [22, 23, 63]. Interestingly, despite the massive increase in reverse transcripts, our data show that HIV-1 has a more modest increase in productive infection after SIVwt pre-infection (Figure 3C). We believe the elimination of SAMHD1 boosts HIV-1 (or RT-HSIV) cDNA generation to supraphysiological levels, which are not needed for HIV-1 to productively infect macrophages, as evidenced by Gag- and GFP-positivity (Figures 1C, 1G, 3C, and 6D). Therefore, although it has been previously shown that SAMHD1 affects HIV-1 infection, we argue that SAMHD1 elimination is not necessary for HIV-1 (or RT-HSIV) to reverse transcribe in macrophages; Vpx and SAMHD1 knockdown only serve to boost HIV-1 infectivity but are not necessary for it.

## DISCUSSION

Myeloid cells pose several obstacles to infection by primate lentiviruses. As terminally differentiated, non-dividing cells, the nuclear envelope would be expected to limit nuclear translocation of the viral reverse transcription or pre-integration complex. Nevertheless, primate lentiviruses have evolved a mechanism, still poorly understood, that allows the reverse transcription complex to gain nuclear access and drive integration within host cell DNA [64]. Secondly, dNTPs, which are the substrates for reverse transcription and cDNA generation, are rate limiting in myeloid cells due to SAMHD1 activity. Nevertheless, primate lentiviruses have evolved to circumvent this potent restriction which enables infection of macrophages and quiescent CD4+ T cells [1, 65]. While the mechanism for SAMHD1 evasion employed by SIV and HIV-2 centers on the Vpx protein, it is not known how HIV-1, which does not harbor a Vpx-like activity, evades SAMHD1 during macrophage infection.

Our study reveals the following main points:

- HIV-1 efficiently infects macrophages in the face of SAMHD1 restriction. Macrophages that are restricted to infection by Vpx-deleted SIV, nevertheless remain permissive to HIV-1 infection
- HIV-1 does not modulate the antiviral environment created by SAMHD1 such that HIV-1-infected macrophages remain restricted to infection by Vpx-deleted SIV.
- In contrast to what has been reported in the literature, the lower sensitivity of HIV-1 to SAMHD1-mediated restriction is not dictated by reverse transcriptase. Therefore, inserting the HIV-1 RT into SIV did not confer reduced SAMHD1 sensitivity to the chimeric SIV.
- A chimeric HIV-1 containing an SIV RT exhibited some capacity to synthesize cDNA in the face of SAMHD1 restriction suggesting that a determinant in the HIV-1 genome, that is outside the RT region, may be involved in the reduced sensitivity of HIV-1 to SAMHD1 restriction.

Together, these data suggest that even though SAMHD1 has some effect on HIV-1, the HIV-1 genome harbors a determinant(s) that underscores its ability to infect macrophages in the face of active SAMHD1. This reveals a fundamental difference between HIV-1 and its relatives HIV-2 and SIV, which are unable to infect macrophages unless SAMHD1 is eliminated and dNTP concentrations are increased to levels suitable for reverse transcription of viral cDNA.

Previous reports have indicated that the reverse transcriptases of HIV-1 and SIV differ in several different kinetic parameters including fidelity and processivity [37, 40, 66–68]. Some studies have demonstrated that HIV-1 reverse transcriptases have a lower Km for dNTPs than those of SIV, allowing them to catalyze cDNA synthesis at low dNTP levels, thereby negating the need to eliminate SAMHD1 altogether [40]. To test this, we replaced the reverse transcriptase of SIV with that of HIV-1 (Figure 4) and tested its ability to reverse transcribe in the presence or absence of Vpx. Surprisingly, RT-SHIV, which contains HIV RT in an SIV backbone, was only able to reverse transcribe when Vpx was present (Figure 5). In other words, the RT-SHIV behaved much like wild-type SIV in that it was dependent on Vpx-mediated SAMHD1 elimination for infection of macrophages. The reciprocal experiment further bolsters this idea because RT-HSIV, which contains SIV RT in an HIV-1 backbone, was still able to reverse transcribe in macrophages without any Vpx activity (Figure 6). This suggests that intrinsic differences in SIV and HIV-1 reverse transcriptases do not explain the ability of HIV-1 to infect macrophages without Vpx. Rather, it points to a determinant within the HIV-1 genome, apart from RT, that dictates the resistance of HIV-1 to SAMHD1 restriction.

There is the possibility that another viral accessory protein (Vif, Vpr, Vpu, or Nef) may contribute to SAMHD1 evasion by HIV-1. Vpr, which is the Vpx paralog in HIV-1, has not been proven to be necessary for HIV-1 or SIV infection of macrophages [69, 70]. However, due to its close similarity to Vpx, Vpr could have some other modulating functions of the cellular milieu that work in concert with other viral proteins to promote infection. We previously demonstrated that HIV-1 *vpr* appears incapable of substituting for *vpx* in the SIV genome [19]. One could hypothesize that there is some other restriction factor besides SAMHD1 that is targeted by both Vpx and HIV-1. For example, 1 study found that a Vpx mutant incapable of SAMHD1 degradation was still able to boost lentiviral infection in non-permissive cells [63]. However, because SAMHD1 knock-down creates permissive conditions for SIVΔvpx infection, it appears that SAMHD1 is the key factor blocking SIVΔvpx infection as well as the main target for Vpx (Figure 2). The inability of HIV-1 to create a permissive environment for SIVΔvpx infection of macrophages, is further demonstrated in the current study where we show that infection with HIV-1 did not render macrophages permissive to infection by SIVΔvpx (Figure 3). At face value, this indicates that the HIV-1 genome does not in fact, harbor a determinant that helps nullify the antiviral conditions created SAMHD1. However, those experiments rely on such a factor acting *in trans* and do not rule out the possibility that the determinant in the HIV-1 genome may act *in cis* to permit reverse transcription in the hostile dNTP environment created by SAMHD1 expression.

Our study further argues against any modifying effect of HIV-1 on SAMHD1 levels. HIV-1 did not alter levels of SAMHD1 or dNTPs in our study (Figure 1). In addition, although the phosphorylation status of SAMHD1 has been shown to affect its ability to restrict infection [13, 71, 72], our preliminary data do not show any change in phosphorylation status after infection by HIV-1 (data not shown). Not affecting SAMHD1 may even be an adaptation by HIV-1 to avoid detection by of innate immunity. With less reverse transcription occurring, HIV-1 would not trigger certain cytosolic DNA sensors like cGAS [73–76]. Despite these reports however, we observed that macrophages that are permissive to HIV-1 remain non-permissive to super-infection by SIVΔvpx which demonstrates that the antiviral environment created by SAMHD1 is not modulated by HIV-1 (Figure 3). Instead, our data indicate that HIV-1 retains the capacity to infect macrophages in the face of SAMHD1 restriction because it is incompletely suppressed by the antiviral conditions created by SAMHD1. Further studies will be needed to identify and characterize the mechanism underscoring the resistance of HIV-1 to the SAMHD1 antiviral environment.

## Figures and Tables

**Supplemental Figure.**
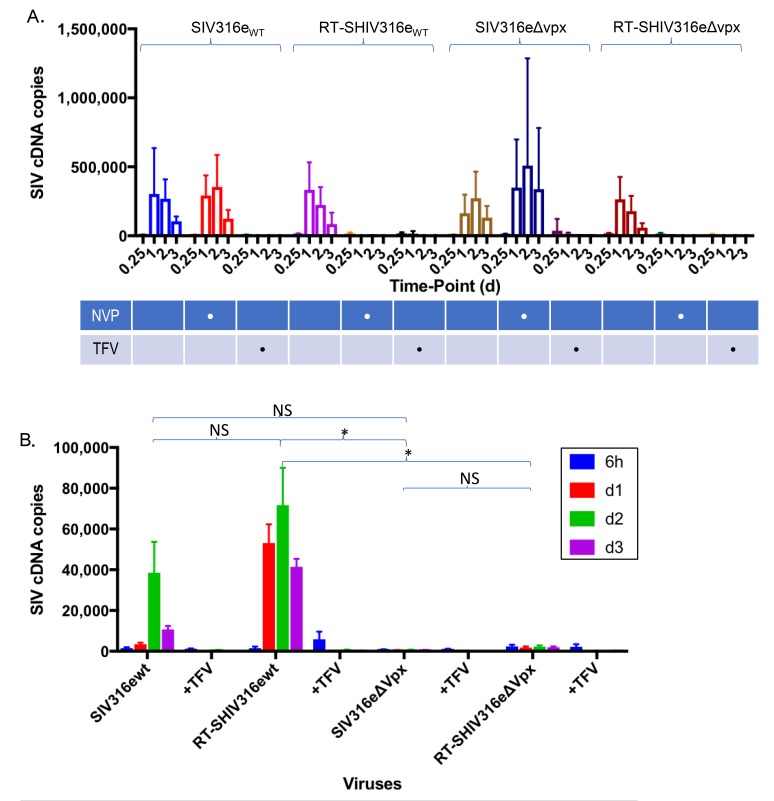
**HIV-1 RT, within the context of an SIVmac316e backbone, requires Vpx for reverse transcription in macrophages.** (A) Differential sensitivity of RT-SHIV316e to the RT inhibitors nevirapine (NVP) and tenofovir (TFV) was assessed in sMAGI cells (susceptible to infection by HIV-1 and SIV). Tenofovir is active against HIV-1 and SIV RT while nevirapine is only active against HIV-1. sMAGI cells were pretreated with the RT inhibitors and then infected with wild type or *vpx*-deleted SIVmac316e variants or with RT-SHIVmac316e variants harboring a functional (RT-SHIVwt) or inactive *vpx* (RT-SHIVΔvpx) open reading frame. Infection of sMAGI cells was gauged from levels of viral cDNA by qPCR at the indicated intervals post-infection. Pooled data from 3 or more replicate experiments +/− SD. (B) Infection of macrophages by SIV harboring an SIVmac316e RT is absolutely dependent on Vpx. Macrophages were infected with RT-SHIV316e variants containing intact (RT-SHIV316ewt) or defective (RTSHIV316eΔvpx) *vpx* open reading frames and infection gauged by qPCR for viral cDNA at the indicated intervals post-infection. Bar graphs in panel A represent pooled data from 3 or more replicate experiments +/− SD. Statistical comparisons for panel B were made from Area Under the Curve analysis. *P < 0.05 **P < 0.01 ***P < 0.001 ****P < 0.0001.
